# Kadish C/Hyams II Esthesioneuroblastoma With Atypical Immunophenotype Mimicking NK/T-Cell Lymphoma: Endoscope-Assisted Transcranial Resection and Adjuvant Intensity-Modulated Radiotherapy

**DOI:** 10.7759/cureus.95351

**Published:** 2025-10-24

**Authors:** Cristina Vanessa Cuevas Calla, Antonio de Jesús González Luna, Nancy Cristina Vela Moya, Marco Antonio Castellanos López, Guadalupe Conejo Flores, Valeria Abril Marcos Rosas

**Affiliations:** 1 Department of General Surgery, Regional Hospital “Dr. Valentin Gomez Farias”, Institute of Security and Social Services for the State Workers (ISSSTE), Zapopan, MEX; 2 Department of General Surgery, University of Guadalajara, Zapopan, MEX; 3 Department of General Surgery, National Polytechnic Institute, Mexico City, MEX; 4 Department of General Surgery, Autonomous University of Guadalajara, Zapopan, MEX; 5 Department of General Surgery, Puebla University Hospital, Puebla, MEX

**Keywords:** esthesioneuroblastoma, olfactory neuroblastoma, paranasal sinus neoplasms, skull base neoplasms, transcranial endoscopic approach

## Abstract

We present a case of an esthesioneuroblastoma (ENB; olfactory neuroblastoma) with an atypical immunophenotype managed by a combined transcranial, endoscope-assisted resection, illustrating diagnostic challenges and surgical decision-making when the anterior skull base and orbit are involved. A 66-year-old woman had recurrent left epistaxis, progressive unilateral nasal obstruction, periorbital pain radiating to the auricle, and ipsilateral visual decline. Examination showed swelling and tenderness over the left nasal region with no lymphadenopathy. Computed tomography (CT) and magnetic resonance imaging (MRI) demonstrated a left sinonasal mass extending to the medial orbit, with erosion of the lamina papyracea and cribriform plate, abutment of the anterior skull base, and molding of the medial rectus without definite intraconal invasion. The initial biopsy suggested natural killer/T-cell (NK/T-cell) lymphoma. Targeted immunohistochemistry (IHC) showed CD56 positivity with a sustentacular S100 pattern, a Ki-67 index of 30%-40%, and negativity for CD3, CD20, granzyme B, T-cell intracellular antigen-1 (TIA-1), chromogranin, and synaptophysin, confirming Kadish C/Hyams II ENB. A bifrontal, endoscope-assisted craniectomy achieved gross total resection and multilayer reconstruction (onlay dural collagen matrix; titanium mesh and polymethylmethacrylate (PMMA) for the anterior skull base; titanium mesh for the orbital roof). Postoperative CT showed no gross residual mass, decompression of the medial rectus, and minimal expected pneumocephalus. Adjuvant intensity-modulated radiotherapy (IMRT) to the surgical bed/anterior skull base was delivered (60 Gy in 30 fractions); elective neck irradiation was individualized. The patient remains stable under multidisciplinary surveillance. Key lessons include broadening the IHC panel to avoid misclassification as NK/T-cell lymphoma when classic neuroendocrine markers are negative, considering a transcranial, endoscope-assisted approach to obtain margins and a stable reconstruction when the anterior skull base/orbit are involved, and obtaining early postoperative imaging as a baseline for surveillance.

## Introduction

Esthesioneuroblastoma (ENB) is a rare malignant neoplasm of the olfactory epithelium, accounting for approximately 3%-6% of sinonasal tumors and presenting with metastatic disease in 20%-48% of cases. Typical metastatic sites include the cervical lymph nodes (10%-33%), bone, and lung; the most common age at presentation is 50-60 years, with a five-year overall survival of 70%-90% and a median time to recurrence of 64 months after initial therapy [1,2]. The presentation is often nonspecific and overlaps with other small round cell tumors, creating diagnostic and therapeutic challenges that require multidisciplinary management [1-3]. Contemporary series describe unilateral nasal obstruction, epistaxis, and hyposmia/anosmia, with many patients diagnosed at locally advanced stages after a median 6-12-month symptomatic intervals [1-3]. Treatment has evolved toward endoscopic resection in selected confined disease. When the anterior skull base, dura mater, or orbit is involved, a transcranial, endoscope-assisted approach (e.g., bifrontal craniectomy) optimizes exposure, permits oncologic margins, and enables a stable reconstruction; adjuvant radiotherapy is recommended for higher stage/grade disease or close/positive margins [1,3-5]. Despite multimodal therapy, late recurrences (cervical, intracranial, distant) occur, necessitating long-term surveillance [4-6]. Our patient had Kadish C/Hyams II ENB involving the anterior skull base and medial orbital wall, treated by bifrontal craniectomy with intracranial endoscopy and complex dural/osseous reconstruction. The initial biopsy suggested natural killer/T-cell (NK/T-cell) lymphoma; a targeted immunohistochemistry (IHC) panel (CD56 and sustentacular S100 positive, Ki-67 30%-40%, lymphoid/cytotoxic markers negative) established the diagnosis. Integrating morphology, an expanded IHC panel, and Kadish/Hyams staging allows timely selection of transcranial endoscopic surgery when intracranial and sinonasal extension coexist and supports risk-adapted adjuvant therapy and follow-up [2,4-6].

## Case presentation

We report the case of a 66-year-old Mexican woman, retired and single, with type 2 diabetes mellitus and systemic hypertension, who developed recurrent left-sided epistaxis, progressive unilateral nasal obstruction, periorbital pain radiating to the auricle, and ipsilateral visual decline in June 2024. She denied tobacco, alcohol, or illicit drug use and reported a diclofenac allergy (rash). Family history included maternal diabetes and lung cancer and paternal prostate cancer, hypertension, and Parkinson’s disease. She lived in an urban setting, followed a balanced diet, and had a complete vaccination schedule, including three COVID-19 doses.

On admission, she was alert, cooperative, and hemodynamically stable. Examination showed swelling and tenderness over the left nasal region without palpable lymphadenopathy; cardiopulmonary, abdominal, and neurological findings were normal. Baseline laboratory testing showed moderate anemia (hemoglobin (Hb), 10.3 g/dL (12.6-16.6); hematocrit (Hct), 32.8% (36.6-47.3); red blood cells (RBCs), 3.65 × 10⁶/µL (4.20-5.40)), with mildly increased blood urea nitrogen (BUN; 27 mg/dL (6-20)), lactate dehydrogenase (LDH; 234 U/L (135-214)), and lactate (1.40 mmol/L (0.30-0.70)). Carcinoembryonic antigen (CEA) was 1.4 ng/mL (0-3 in non-smokers/0-5 in smokers); other tumor markers were within reference limits. All remaining parameters were within or near reference ranges.

**Figure 1:**
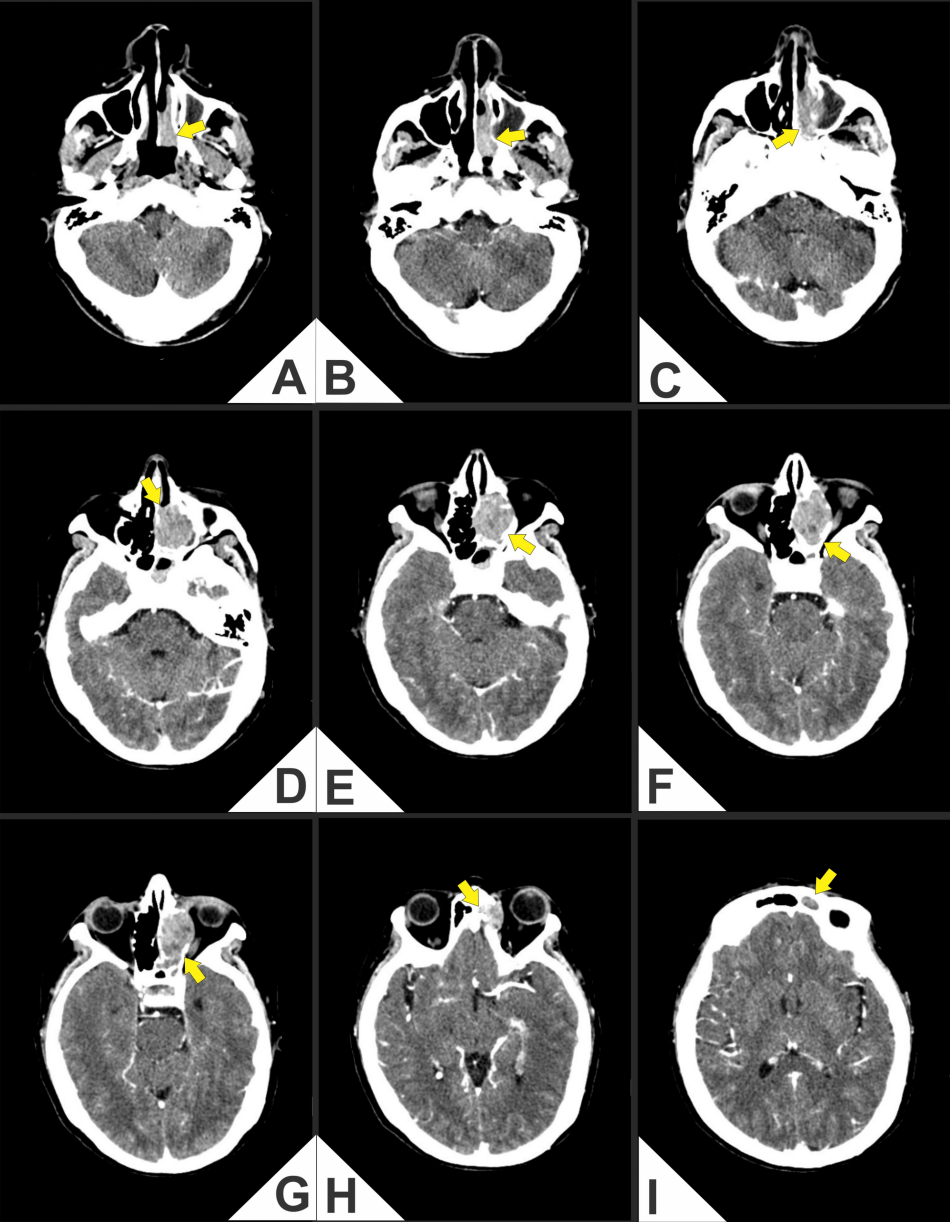
(A-I) Contrast-enhanced axial computed tomography (CT) of the paranasal sinuses and anterior skull base. Heterogeneously enhancing soft-tissue mass centered in the left nasoethmoid-maxillary complex (arrows) with hypodense areas, causing remodeling and erosion of the lamina papyracea and extension to the medial orbit with contact and displacement of the medial rectus muscle, accompanied by effacement of the extraconal fat plane and no definite intraconal involvement (D-G). On more cranial sections (H, I), cortical irregularity and probable dehiscence of the ethmoid roof/cribriform plate region are identified, suggesting anterior skull base involvement. Reactive opacification of ethmoid air cells and adjacent paranasal sinuses is present.

**Figure 2:**
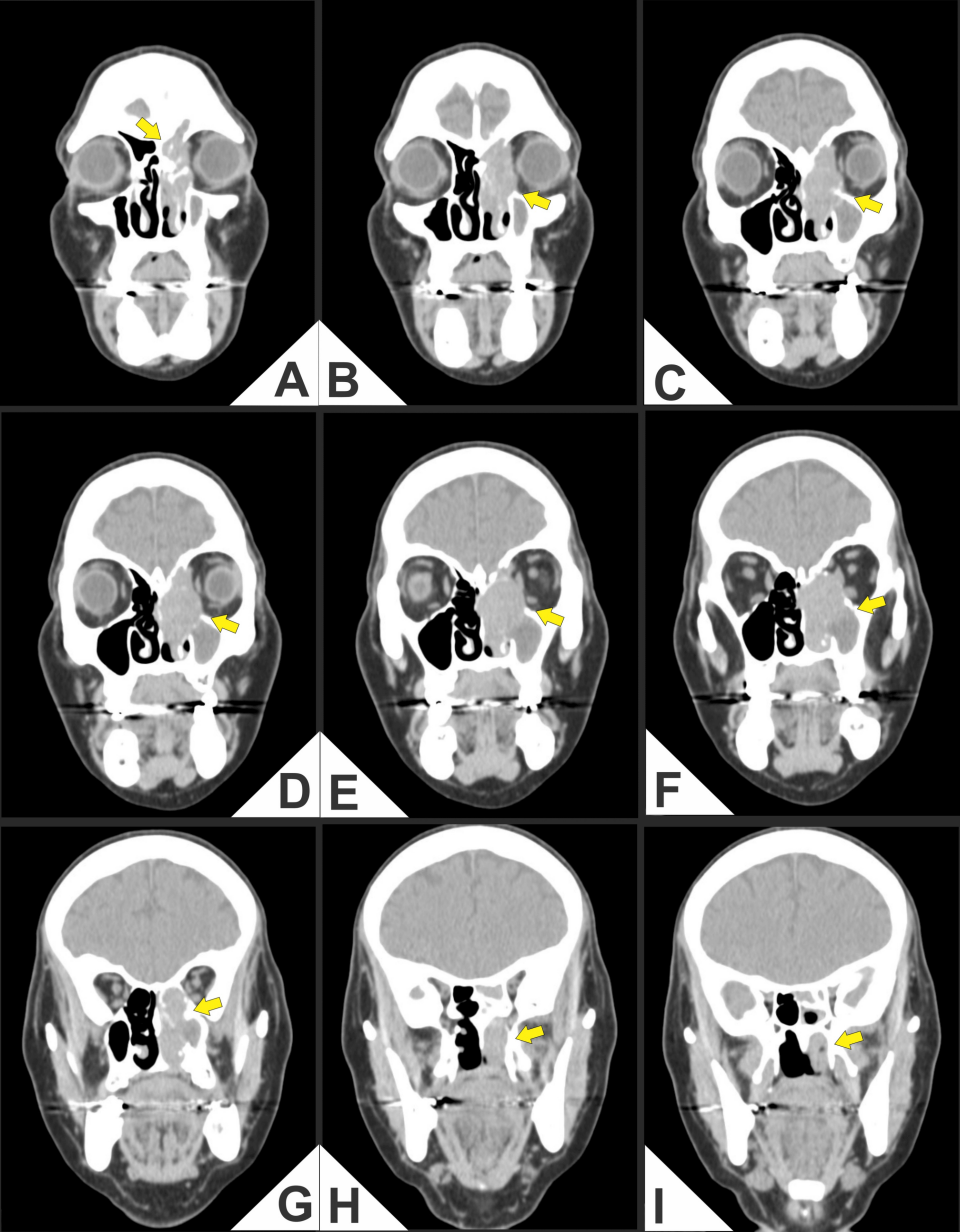
(A-I) Coronal computed tomography (CT; soft-tissue window throughout) of the paranasal sinuses and anterior skull base. Heterogeneously enhancing soft-tissue mass centered in the left nasoethmoid-maxillary complex (arrows). (A) Cortical irregularity and a focal defect of the fovea ethmoidalis/cribriform plate, suggesting anterior skull base involvement. (B, C) Tumor filling the left anterior ethmoid air cells with dehiscence of the lamina papyracea and medial extraconal orbital extension, characterized by contact and flattening of the medial rectus muscle and effacement of the extraconal fat plane, without definite intraconal invasion. (D-F) Persistent heterogeneous enhancement with internal hypodense areas and continued erosion of the medial orbital wall; the globe contour and attenuation are preserved. (G-I) Near-complete opacification of the left maxillary sinus with remodeling/erosion of its medial wall and subtle fullness/stranding in the left pterygopalatine fossa, suggesting early posterior extension.

**Figure 3:**
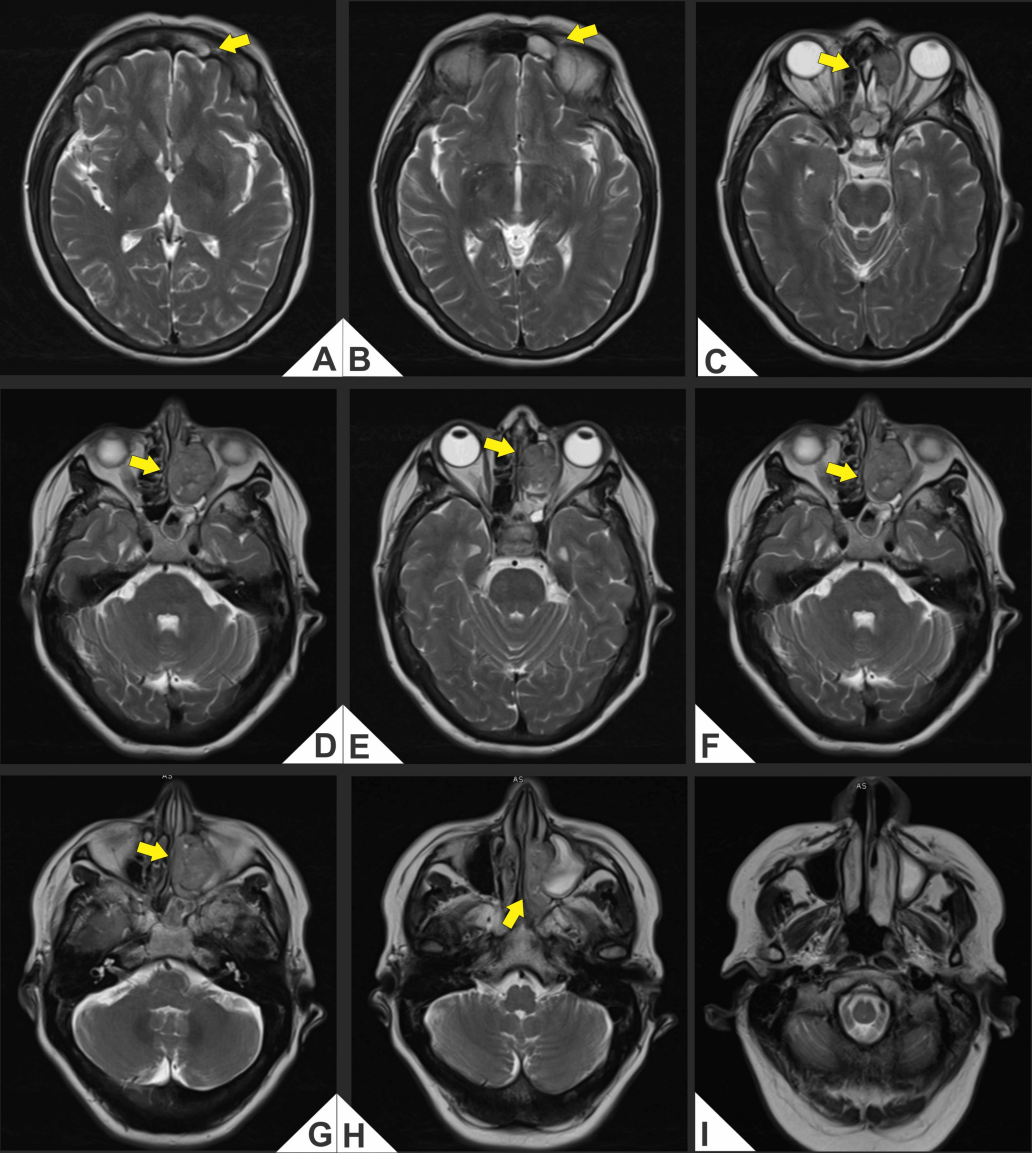
(A-I) Axial T2-weighted magnetic resonance imaging (MRI; non-fat-suppressed). Lobulated T2-hyperintense mass centered in the left nasoethmoid region, measuring 3.24 × 2.47 × 4.22 cm (craniocaudal × anteroposterior × transverse), with medial extraconal orbital extension that molds the medial rectus and effaces the adjacent extraconal fat, without definite intraconal involvement. (A, B) Effacement of the subfrontal fat plane with abutment of the cribriform plate/anterior skull base; (C-H) Persistent heterogeneous T2 signal with internal hypointense foci and inferior extension toward the ethmoid-meatal recess and the medial wall of the left maxillary sinus. Findings are concordant with Kadish C topography and with lamina papyracea erosion documented on CT (Figure 1 and Figure 2).

**Figure 4:**
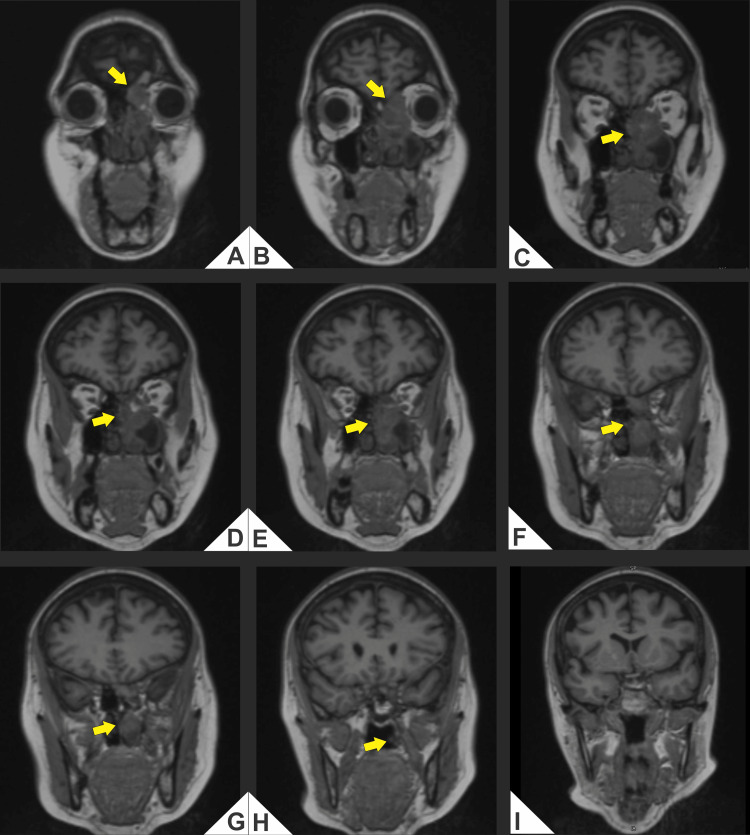
(A-I) Coronal T1-weighted magnetic resonance imaging (MRI; non-fat-suppressed, non-contrast). Left nasoethmoid soft-tissue mass with low-to-intermediate T1 signal (arrows) abutting the ethmoid roof/cribriform plate and causing effacement of the subfrontal fat plane, compatible with anterior skull base contact. There is medial extraconal orbital extension with molding and lateral displacement of the medial rectus and loss of the adjacent extraconal fat plane, without definite intraconal or optic nerve involvement in this series. Inferior extension toward the middle meatus and the medial wall of the left maxillary sinus is noted; scattered relatively hyperintense foci may reflect blood/proteinaceous content.

Computed tomography (CT) and magnetic resonance imaging (MRI) demonstrated an aggressive mass in the left nasal cavity and paranasal sinuses with medial orbital extension and erosion of the cribriform plate and lamina papyracea, abutting the anterior skull base (Figures 1-4).

**Figure 5:**
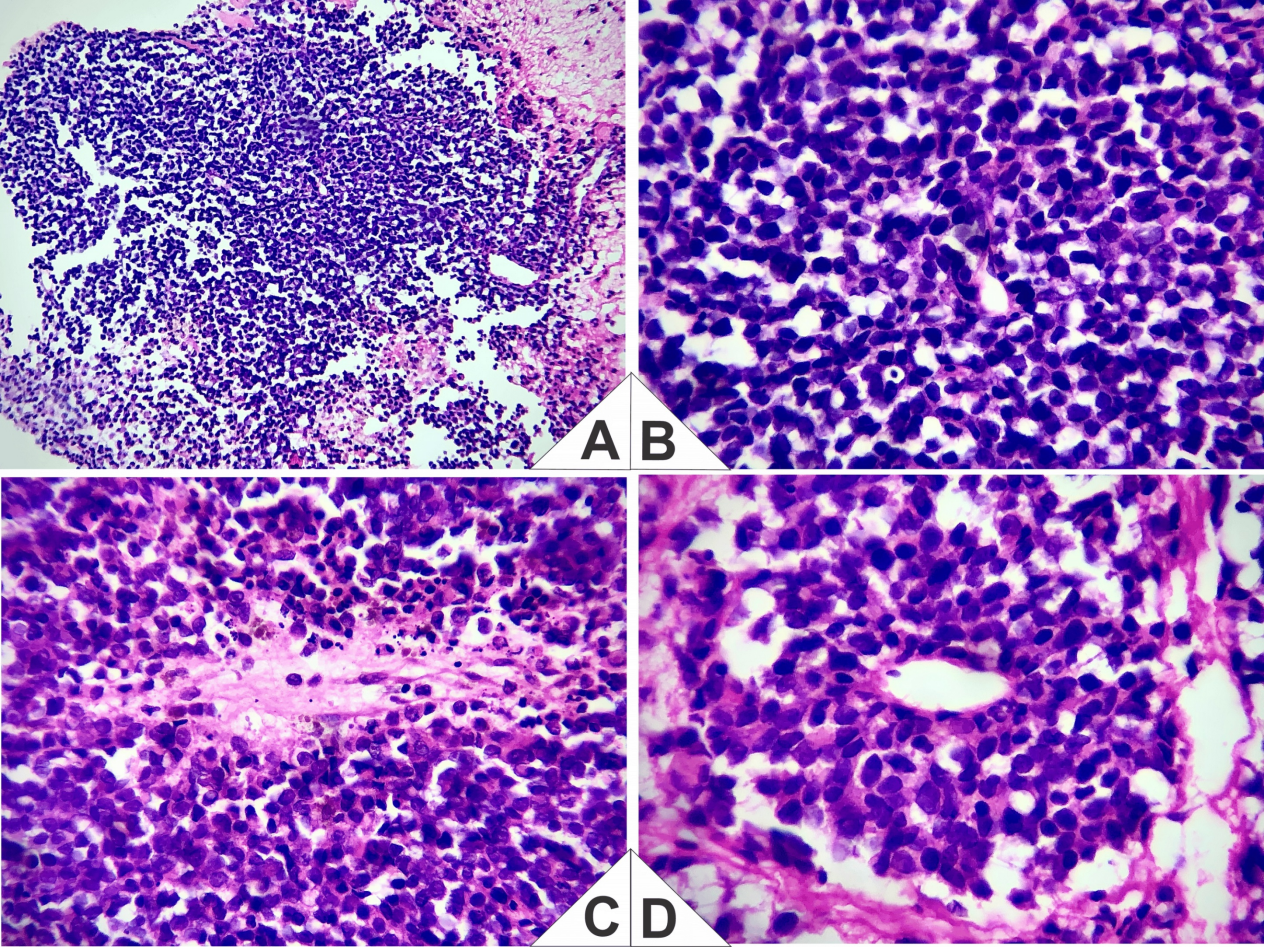
Representative histopathology (hematoxylin and eosin (H&E)). (A) Low power: lobular architecture separated by delicate fibrovascular septa with a neurofibrillary/neuropil background. (B) High power: sheets and nests of small round blue cells with scant cytoplasm and fine (“salt-and-pepper”) chromatin; scattered mitotic figures and apoptotic bodies. (C) Homer-Wright pseudorosettes: radial palisading around an eosinophilic fibrillary center (neuropil) without a true lumen. (D) True rosette with a central lumen, Flexner-Wintersteiner-like, lined by more columnar, apically oriented cells showing nuclear palisading. Taken together, and in correlation with the immunophenotype (CD56 positive, sustentacular S100; Ki-67 30%-40%; negative CD3, CD20, TIA-1, granzyme B, chromogranin, and synaptophysin), the findings support Hyams Grade II esthesioneuroblastoma.

**Figure 6:**
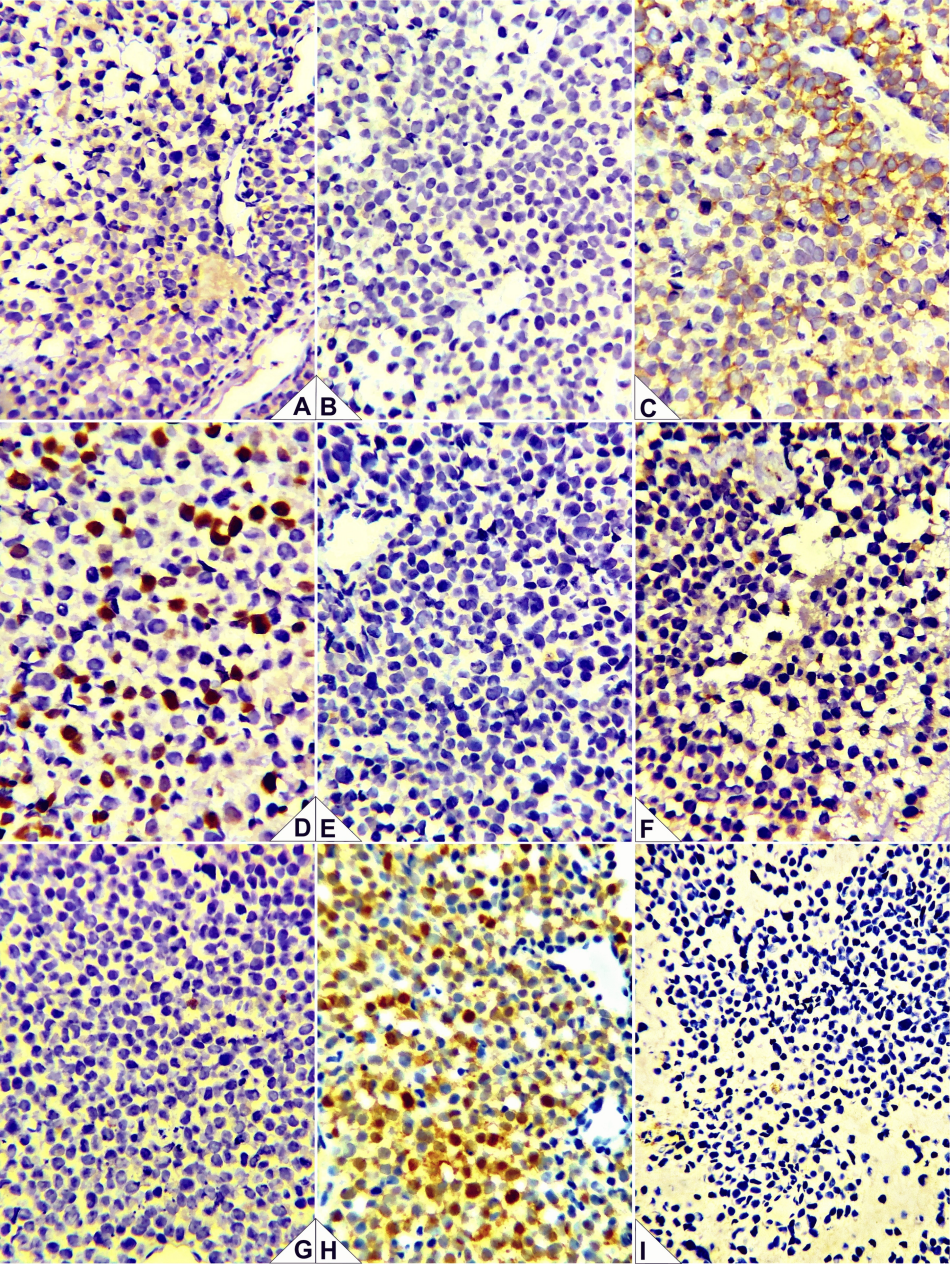
Immunohistochemistry (IHC) panel. (A) CD3: negative in neoplastic cells (appropriate internal/external controls). (B) CD20: negative in neoplastic cells (controls adequate). (C) CD56: diffuse membranous/cytoplasmic positivity in tumor cells. (D) Ki-67: proliferation index ~40% in hot spots. (E) Chromogranin A: negative. (F) Granzyme B: negative. (G) TIA-1: negative. (H) S100 protein: sustentacular pattern highlights peripheral sustentacular cells encasing tumor nests. (I) Synaptophysin: negative. Interpretation: The combination of CD56 positivity with a sustentacular S100 pattern and absence of B-/T-cell and cytotoxic markers (CD3, CD20, TIA-1, granzyme B), together with the lack of classic neuroendocrine markers (chromogranin/synaptophysin) and the histomorphology, supports Hyams Grade II esthesioneuroblastoma and argues against extranodal NK/T-cell lymphoma.

The initial biopsy reported a poorly differentiated malignant neoplasm with a differential diagnosis of nasal NK/T-cell lymphoma versus ENB. Slide review with a targeted IHC panel showed CD56 positivity with a sustentacular S100 pattern, Ki-67 30%-40%, and negativity for CD3, CD20, chromogranin, granzyme B, TIA-1, and synaptophysin (Figure 5 and Figure 6).

Clinicoradiologic-morphologic correlation (diagnostic justification)

The topography centered on the olfactory cleft/anterior skull base with cribriform and lamina papyracea erosion and medial orbital extension is characteristic of ENB. On H&E, there were lobular architecture, a neurofibrillary matrix, and rosettes/pseudorosettes (Homer-Wright and Flexner-Wintersteiner), with mild-to-moderate atypia, scant mitoses, and no extensive intratumoral necrosis, consistent with Hyams II. On IHC, CD56 positivity and a peripheral sustentacular S100 pattern (with Ki-67 30%-40%), together with negativity for CD3, CD20, TIA-1, granzyme B, chromogranin, and synaptophysin, support ENB and argue against NK/T-cell lymphoma (absence of lymphoid/cytotoxic markers) and undifferentiated/neuroendocrine carcinomas (given the neurofibrillary matrix and the sustentacular S100 pattern). In aggregate, the integration of clinical findings, imaging, morphology, and IHC was sufficient to support the final diagnosis of ENB (Kadish C/Hyams II).

**Figure 7:**
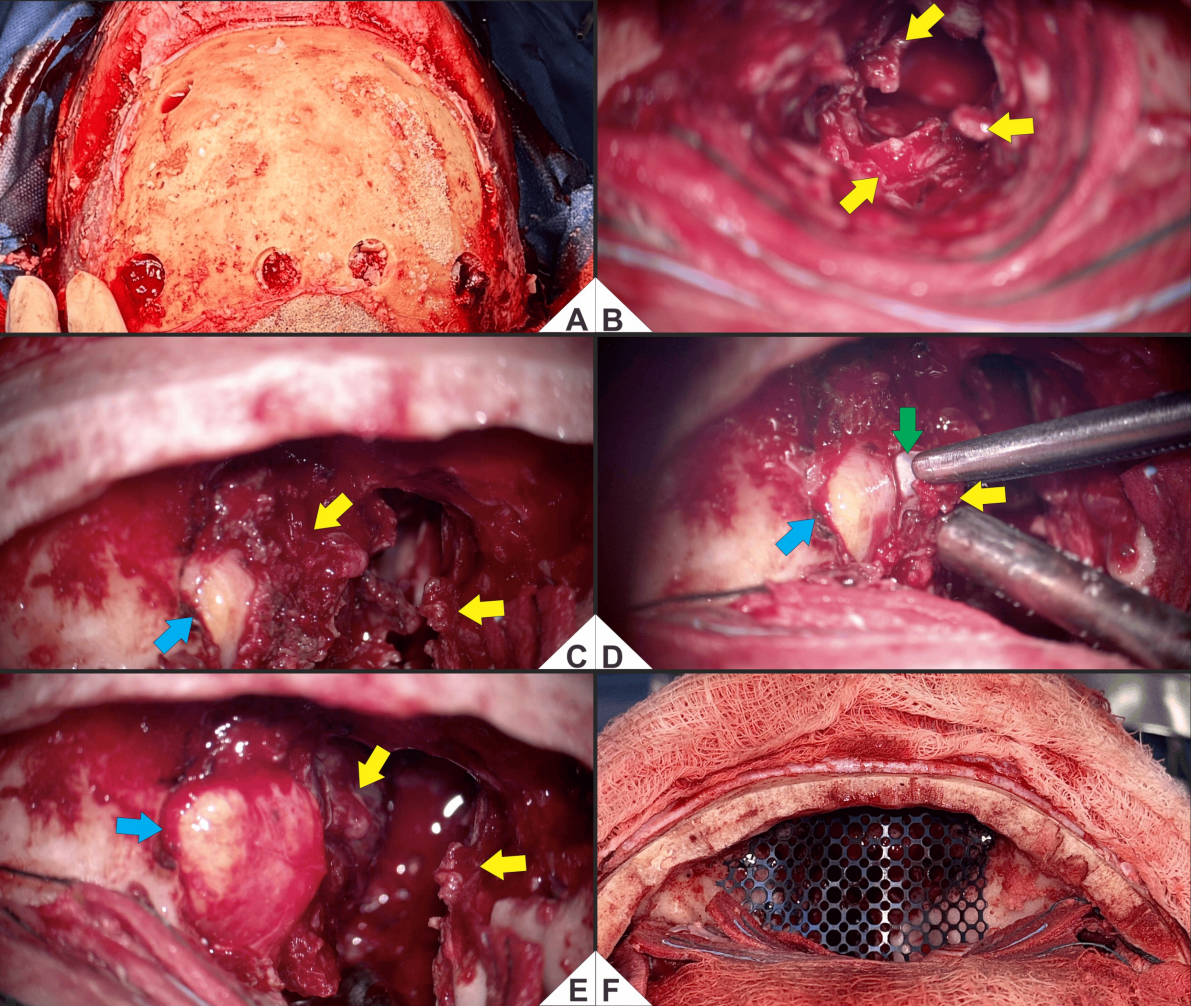
(A-F) Endoscope-assisted transcranial resection and anterior skull base reconstruction. (A) Pre-craniectomy exposure after bicoronal incision; scalp-galea elevated and pericranial flap preserved for reconstruction. Four burr holes mark the planned bifrontal osteotomy. (B) After initial bone removal, a lobulated, friable, vascular tumor occupies the left nasoethmoid-maxillary region (yellow). (C) Medial orbital wall exposure: tumor abuts the periorbita with a medial extraconal bulge; the globe remains intact behind the periorbita (blue). (D) Subperiosteal dissection defines a clear cleavage plane between tumor capsule and periorbita/globe (green); debulking proceeds with globe preservation (blue) and circumferential release of the mass (yellow). (E) After circumferential mobilization, irregular residual nodules persist in the bed during controlled resection (yellow); the globe remains protected (blue). (F) Anterior skull-base reconstruction: a contoured microperforated titanium mesh bridges the defect; onlay dural repair with collagen lies beneath (not shown). The implant shows stable seating and hemostasis; the construct is reinforced with in situ-molded PMMA to re-establish cranio-nasal separation. Color code: yellow = tumor; blue = globe; green = tumor-periorbita cleavage plane. Technical note: Resection used blunt-sharp subperiosteal dissection with sequential debulking and medial orbital control, enabling macroscopic clearance of sinonasal disease and multilayer reconstruction of the anterior skull base.

Given these findings, surgery was pursued. On August 22, 2025, the patient underwent a bifrontal craniectomy with transcranial oncologic resection assisted by intracranial endoscopy (non-endonasal) (Figure 7).

Intraoperatively, there was involvement of the dura, ethmoid bone, sphenoid and maxillary sinuses, and orbital roof, with partial involvement of the medial rectus muscle and periorbital fat. An oncologic resection was performed, achieving gross total removal across involved sinonasal and intracranial compartments (Figures 7B-7E), followed by multilayer reconstruction: onlay dural repair with DuraGen® (Integra LifeSciences, Princeton, NJ, USA), anterior skull base reconstruction with titanium mesh and polymethylmethacrylate (PMMA), and orbital roof reconstruction with titanium mesh. Osteosynthesis was achieved with miniplates, and a subgaleal drain was placed (Figure 7F).

**Figure 8:**
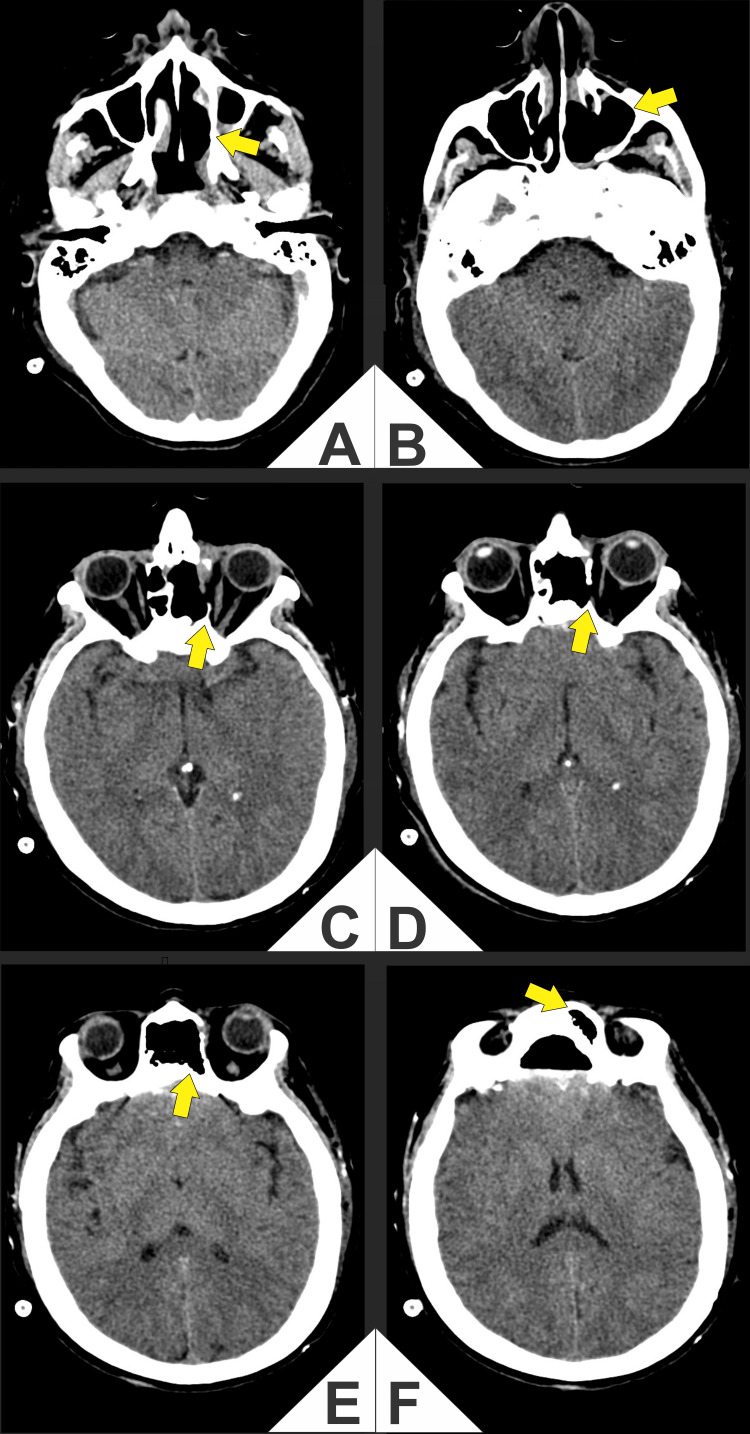
(A-F) Early postoperative axial computed tomography (CT). Arrows indicate the previous tumor bed in the left nasal cavity/ethmoid and medial orbital wall. Postoperative changes after bifrontal craniectomy and endoscope-assisted transcranial resection include anterior skull base reconstruction (titanium mesh with polymethylmethacrylate cranioplasty) and decompression of the medial rectus with reconstitution of the medial extraconal fat plane, with no evident residual mass. Reactive sinonasal opacification and minimal pneumocephalus/subgaleal air are expected in the early postoperative period.

Early postoperative CT was obtained to verify oncologic resection, assess the bony/prosthetic reconstruction (titanium mesh and PMMA), and rule out acute complications. CT showed no gross residual solid mass at the prior tumor bed (arrows), decompression of the left medial rectus with restoration of the extraconal fat plane, a controlled postsurgical defect of the lamina papyracea, and a regular anterior skull base reconstruction, with minimal expected pneumocephalus and reactive sinonasal opacification, and no intracranial or orbital collections (Figure 8).

The immediate postoperative course was uneventful. She was discharged on postoperative day 3 with preserved extraocular movements and no new neurologic deficits (ECOG performance status 0; Karnofsky Performance Status 90-100). Final histopathology confirmed ENB, Hyams Grade II. Based on resection status and imaging, the patient received adjuvant intensity-modulated radiotherapy (IMRT) to the surgical bed and anterior skull base (60 Gy in 30 fractions), with individualized elective cervical irradiation. Acute toxicities were limited to Grade 1-2 dermatitis, nasal dryness/rhinitis, and fatigue; there were no visual disturbances, cerebrospinal fluid leaks, or infections. Nasal endoscopy demonstrated a well-healed cavity without residual mass. Neck examination and ultrasound showed no nodal disease. At the most recent visit, one month postoperatively, the patient remained clinically disease-free, with preserved visual function and normal daily activities. The surveillance plan includes MRI of the anterior skull base every 4-6 months for the first two years, annual chest imaging, and endoscopic evaluation per multidisciplinary protocol, with subsequent extension of intervals.

## Discussion

Contemporary series consistently report that ENB presents at locally advanced stages and that five-year overall survival approximates 70%-90%; our Kadish C/Hyams II case with anterior skull base contact and medial orbital extension conforms to that distribution and clinical behavior [1-3]. This alignment likely reflects the same anatomic pattern, cribriform plate and lamina papyracea erosion with medial orbital abutment, described across those cohorts [1-3]. Diagnostic classification required integrating morphology and IHC because classical neuroendocrine markers may be negative in a subset of tumors. Our profile, CD56 positivity with a sustentacular S100 rim and Ki-67 around 30%-40% but negative synaptophysin/chromogranin, falls within the recognized immunophenotypic spectrum; in such settings, insulinoma-associated protein 1 (INSM1) improves diagnostic sensitivity and has been associated with inferior disease-free and overall survival in recent cohorts, supporting its use when classical markers are absent [7-10]. The negative lymphoid/cytotoxic panel and lack of Epstein-Barr virus-encoded RNA (EBER) signal argued against NK/T-cell lymphoma, consistent with recommended algorithms that combine topography at the olfactory cleft/anterior skull base, morphology (lobular architecture, neurofibrillary matrix, rosettes), and a targeted IHC panel [7,8]. Management decisions mirrored current evidence. Although endoscopic surgery is favored for less advanced disease, open or transcranial, endoscope-assisted approaches remain appropriate when the anterior skull base, dura, or orbit is involved to secure margins and permit a stable multilayer reconstruction; our choice of a bifrontal, endoscope-assisted resection therefore converged with risk-adapted practice in anatomically complex Kadish C tumors [1,3,7,11]. Adjuvant IMRT to the surgical bed is standard in higher stage/grade disease or when margins are close/positive and was used here to consolidate local control [1,3-5]. Cervical management was individualized in light of a proportion meta-analysis showing a non-trivial but heterogeneous prevalence of nodal metastasis at presentation and at relapse, supporting selective rather than universal elective neck irradiation in clinically node-negative patients [6]. Finally, because late regional, intracranial, and distant recurrences are well documented, and pathologic dural invasion correlates with regional failure, structured, long-term surveillance is essential [4,5]; our follow-up schedule reflects that risk landscape [4-6]. In sum, our findings are concordant with contemporary cohorts in stage at presentation, diagnostic pathway, treatment sequencing, and surveillance rationale; where our strategy diverges from purely endoscopic options, the difference is explained by cranio-orbital complexity and reconstruction needs, which are precisely the circumstances in which open/hybrid approaches remain indicated [1,3,7,11].

## Conclusions

In this Kadish C/Hyams II ENB, three practical lessons emerged. First, when classical neuroendocrine markers are absent, diagnosis should integrate morphology with a focused IHC panel centered on CD56 and a sustentacular S100 rim, supplemented by INSM1 for sensitivity and exclusion of lymphoma through EBER and cytotoxic markers. Second, anterior skull base and medial orbital extension may favor a transcranial, endoscope-assisted approach to achieve clear margins and enable stable multilayer reconstruction, rather than a purely endoscopic route. Third, adjuvant IMRT supports local control in higher-risk disease, and long-term structured surveillance remains essential, given the potential for delayed regional, intracranial, or distant relapse, particularly when dural invasion is present.
